# Large Vegetations of Mitral Valve—Pneumonia, &c.

**Published:** 1842-05-28

**Authors:** M. W. Wilson

**Affiliations:** Resident Physician


### CLINICAL REPORTS.

### *Blockley Hospital—Service of W. W. Gerhard, M. D.*

Reported by M. W. Wilson, M. D., Resident Physician.

*Large Vegetations of Mitral Valve—Pneumonia, &c.*—George White, æt. 42, a coloured man, entered medical ward May 4th, 1842. Came from Philadelphia; born in Maryland. Taken ill April 26th, but had a cold for a week previous. Was first taken with a sharp pain in the præcordial region, shooting across the sternum while at work; occasionally awakened from sleep with it; cough rare and dry; decubitus dorsal; no chills; fever severe and constant; had hoarseness from commencement of pain. Symptoms have not increased for some days, nor expectoration altered in character. On the evening of entrance took a mustard foot bath, and ten scarified cups applied to chest.

℞. Pulv. Ipecac. ʒj.

Aquæ Bullient. Oj. ℥ij. ter in die.

On the 5th the cups were repeated; about ℥xj. of blood taken in the two applications. Felt easier after the cupping.

6th. *Present state.*—Intellect clear; no cephalalgia; slept well during the fore part of last night; hoarseness; cough tolerably loose; expectoration thick mucus, opaque, rather viscid; decubitus dorsal; respiration frequent, about thirty-six, high. Pulse 112, quick, moderately full; tongue nearly clean; great thirst; no appetite; bowels moved last night. Respiration slightly bronchial in the upper lobe of left lung; subcrepitant rhoncus in lower lobe. Right lung bronchial in upper lobe, deep-seated crepitus in middle lobe, and puerile in lower lobe. Percussion on the upper part of the chest on both sides.

℞. Venesection.

Other treatment continued.

7th. Pulse 120, weak; respiration forty, mostly abdominal; dyspnœa increased; expectoration viscid and of a rust colour. Ordered twelve dry cups over chest, and

℞. Ammon. Muriat. ʒj.

Syr. Senegæ, ℥j.

Mucilag. Gum Acac. q. s. ℥vj.

ℳ. Mag. Coch. q. h. 2.

Evening. Pulse 128. Respiration thirty-two, irregular.

℞. Vini. ℥iv. per diem.

8th. Slept badly; cough troublesome; pulse quick and frequent, not counted; respiration hurried. Continue treatment.

Evening. Pulse 120. Respiration thirty-six. No change.

9th. Pulse 124, feeble. Respiration thirty-six. Percussion as before. Respiration bronchial, but less intense at the summit of both lungs. Increase the wine ℥iv. Other treatment continued.

Evening. Pulse 108. Respiration forty. Subsultus tendinum; eyes glassy; great prostration.

℞. Ammon. Carb. ʒj.

Spts. Æth. Sulph. Comp. ℥ss.

Mucil. G. Acac. q. s. ℥vj.

M. Mag. Coch. q. h. s.

10th. Pulse 140, very feeble. Respiration forty, high and irregular; less subsultus; slept during the latter part of the night. Bowels moved twice. Continue treatment.

11th. Prostration less; still considerable. Pulse 132; extremities cool; no pain; feels extremely weak.

Continue treatment.

12th. Pulse extremely feeble, about 140. Respiration frequent and irregular.

*Heart.**—First sound marked by a strong blowing sound at the apex, and regurgitation with the second. Impulse greater than usual. Continue treatment.

13th. Pulse 128, feeble; extremely weak, scarcely able to expectorate; the effort of expelling sputa from his mouth nearly exhausts him; starts every few minutes as though frightened; respiration frequent and very irregular. Continue treatment.

14th. Pulse 128, feeble. Respiration thirty-two. Subsultus continues; extreme prostration. Continue treatment.

Evening. No change since morning. Died on the morning following.

*Autopsy twenty-seven hours after death.*

*Thorax.*—Extensive adhesions of both pleura.

*Left lung* (upper lobe) much engorged with serum; lower lobe engorged with blood and commencing granulations; no tubercles.

*Right lung.*—Upper lobe indurated; contains no air; on pressure, a dirty yellowish red puriform fluid exudes. Middle lobe, tint less dark; fluid dirty red, less yellowish.

Lower lobe imperfectly granulated, and contains a reddish fluid.

*Heart* much enlarged; semi-lunar valves flexible, only slightly thickened, with one exception. On inner surface of thickened valve the membrane is thickened, and recent lymph deposited. Mitral valve distorted; original form destroyed; one lip shortened and shrunken in part, from recent lymph adhering to its surface, and partly from cartilaginous deposition between its membranes. The other lip forms an irregular mass three-fourths of an inch thick, the internal membrane covering, which is extremely thickened and opaque; but the thickness of the mass is formed in the main by a soft substance, resembling in consistence the fibrine of aneurism diffused between the two membranes. The edges of the mass are knobbed and irregular.

*Right side of heart.*—Valves healthy; lining membrane smooth. Internal membrane of aorta smooth, and nearly natural.

*Abdomen.*—Liver pale; slightly fatty; of normal size.

*Kidneys* exhibits marks of commencing granulations.

In this case the fatal termination depended mainly upon the extent of the lesions in the chest. The alteration of the valves of the heart was in part of long standing, and in part depended upon the acute inflammation of the endocardium, which often accompanies severe pneumonia. There was another cause, however, which was very efficient in bringing about the death of the patient; that was the character of pneumonia during the spring of the present year. Many of the cases were decidedly asthenic, others, if not at first absolutely asthenic, became so under moderate bloodletting; in the case of our patient it could not be borne, or at least produced no beneficial change in the character of the disease.

The practical inferences are very obvious. When the dangerous complication of endocarditis accompanies pneumonia, the disease may still be cured if ac-

* His heart was examined at the time of his entrance, but no note taken of it until this date. The sounds were the same with very little variation at the time of his entrance in the wards.

tive bloodletting be practised, but if the patient has already sunk into the third stage of the disease, or if it has been of the asthenic form from the beginning, our means of treatment fail us. When the patient was placed upon the use of diffusible stimulants he rallied for a moment, but the disorder of the circulation soon increased so much that stimulants completely failed in their influence.

